# *Staphylococcus aureus* bacteriuria: implications and management

**DOI:** 10.1093/jacamr/dlac123

**Published:** 2023-01-11

**Authors:** C Y Mason, A Sobti, A L Goodman

**Affiliations:** Department of Infectious Diseases, Guy’s and St Thomas’ NHS Foundation Trust, Westminster Bridge Road, London SE1 7EH, UK; Department of Infectious Diseases, Guy’s and St Thomas’ NHS Foundation Trust, Westminster Bridge Road, London SE1 7EH, UK; Department of Infectious Diseases, Guy’s and St Thomas’ NHS Foundation Trust, Westminster Bridge Road, London SE1 7EH, UK; Department of Population Health, Medical Research Council Clinical Trials Unit, London, UK

## Abstract

**Background:**

*Staphylococcus aureus* is isolated in around 0.2%–4% of positive urinary cultures, more commonly in the contexts of long-term care, urological abnormalities and procedures, male sex, older age and comorbidities. Isolation may represent contamination, colonization, urinary tract infection or bacteraemic seeding from another site, and may be linked to *S. aureus* bacteraemia. However, there is little guidance on investigation and management of *S. aureus* bacteriuria. We performed a retrospective analysis of cases in our service, including clinical characteristics, investigations and treatment.

**Methods:**

Data were collected on all urine samples taken from adult patients over a 5-year period from which *S. aureus* was isolated. Detailed analysis including investigations and management was conducted in those collected over a 1-year period.

**Results:**

From 511 patients, 668 urine cultures positive for *S. aureus* were identified; 6.5% of cases were positive for MRSA. Of 93 patients who had blood cultures taken, there were 6 cases of *S. aureus* bacteraemia, 4 of which were associated with urological instrumentation. Of 94 cases analysed in detail, 57% were treated with antibiotics, and 49% had repeat urine cultures. Factors associated with recurrence were urinary catheterization, urological abnormality, diabetes and inpatient status.

**Conclusions:**

Our experience does not support the routine taking of blood cultures or treatment of asymptomatic bacteriuria in well patients with *S. aureus* bacteriuria in this setting. However, repeat urine culture, and investigation and treatment of higher risk patients, for example, prior to bladder instrumentation, may be warranted. We propose a simple algorithm to guide clinicians.

## Introduction

*Staphylococcus aureus* is isolated in around 0.2%–4% of positive urinary cultures.^1–4^ It is isolated more commonly in those in long-term care, with long-term catheters, urological abnormalities and procedures,^3^ male sex, older age and comorbidities.^2^ Isolation may represent a wide range of conditions, including contamination, colonization, urinary tract infection or bacteraemic seeding from another site.^5^ Several studies have sought to clarify the relationship between *Staphylococcus aureus* bacteriuria (SABU), *Staphylococcus aureus* bacteraemia (SAB) and invasive staphylococcal disease.^2,6,7^ It is postulated that SAB may be a cause or consequence of SABU, with risk factors for concurrent SAB including male sex, inpatient status, indicators of systemic infection, urinary tract abnormalities, lack of symptoms of urinary tract infection (UTI) and diabetes.^2,3,8,9^ However, there is a paucity of guidance on the investigation and management of SABU,^10^ including optimum antibiotic treatment.

The clinical relevance of SABU is unclear. The rate of associated SAB in patients with SABU has been reported as between 8% and 27%^3,9,11^ and is linked to poorer outcomes.^1^ Arpi and Renneberg^3^ found that in a series of 132 community hospital inpatients with SABU, 8.3% developed SAB. All were thought to have developed SAB secondary to SABU, with urinary catheterization, urological abnormalities and instrumentation being major risk factors for developing SAB.^3^ Conversely, SABU can occur as a result of SAB, and its presence in this context is an independent risk factor for mortality.^12,13^ Al Mohajer *et al.* in a series of 326 patients with SABU, found that in those with MRSA and MSSA SABU, 22% and 8.4% developed SAB within 12 months, respectively. Risk factors for invasive disease include lack of symptoms of UTI and inpatient status.^14^ In only one patient was the source of SAB thought to be urinary. It has been proposed that SABU could be a pointer to invasive disease such as discitis or infective endocarditis, especially in the absence of risk factors for colonization.^15^ In infective endocarditis its presence is predictive of worse outcome, perhaps because it denotes vasculitic spread manifested by features such as renal microabscesses.^6^ It has therefore been suggested that a finding of SABU should prompt further investigation with blood cultures.^8,10^ Although some consider SABU without SAB to represent colonization, *S. aureus* urinary tract infection is also an increasingly recognized entity.^11^ This complex range of possible clinical significance of a finding of SABU, including contamination, colonization, asymptomatic bacteriuria, primary urinary tract infection and manifestation of invasive disease, as well as discrepancies in results of studies of the role of antibiotics in SABU^3,14^ and the importance of antibiotic stewardship, pose a challenge to the clinician in judging which cases to investigate further and to treat.

Our service evaluation describes the characteristics of patients with SABU in a UK tertiary centre, and how they are investigated and managed, with an aim of suggesting how this could be improved. We performed a retrospective analysis of cases of SABU over a 5-year period, including provenance, resistance patterns, and rates of SAB. In a subset of cases from one year we further analysed the investigation and treatment of cases and rates of recurrence. Based on our findings and those of previous studies we propose a simple algorithm for investigation and management of *S. aureus* bacteriuria.

## Patients and methods

### Data collection

Data were collected on all urine samples from which *S. aureus* was isolated taken between 1 March 2016 and 28 February 2021, from adult patients (age ≥18 years). These included the specimen type, setting in which the sample was obtained and susceptibility patterns of the organisms. Data were also collected on blood cultures taken within 3 months of these samples, and any positive blood cultures taken within a year. Where there was more than one positive sample for an individual patient only the first positive sample was included in analysis. For samples collected between 1 March 2020 and 28 February 2021 further data were collected from electronic patient notes including patients’ symptoms, urine microscopy, colony count, recurrence, other cultures positive for *S. aureus* and comorbidities. Symptoms of urinary tract infection were defined as dysuria, suprapubic pain, gross haematuria, urinary frequency or loin pain, or fever or confusion without another cause identified.

### Microbiology

Urine samples (1 µL) were cultured on chromID CPS Elite (bioMérieux, UK) agar for a maximum of 18–24 h. Species were identified using MALDI-TOF (Bruker, Germany). Antimicrobial susceptibility testing was performed using up-to-date BSAC breakpoints (until 5 December 2016) or EUCAST break points (from 6 December 2016) using disc diffusion on Mueller–Hinton agar (Oxoid) and VITEK (bioMérieux, UK)

### Ethics

This was a service evaluation of the impact of the urine results on routinely collected data. We obtained local permission through our service evaluation approval team (internal ref. 10687).

### Statistics

Contingency tables were analysed using the chi-square test.

## Results

We identified 669 urine samples positive for *S. aureus* from 511 individual patients. Characteristics of the patients are shown in Table 1; 6.5% of the isolates (*N* = 511) were MRSA.

**Table 1.** Demographic characteristics of patients with *S. aureus* bacteriuria SABU

	All cases (2016–21) *N* = 511	Cases studied in further detail (2020–21) *n* = 100
Age (years)	Mean 54, range 18–96	Mean 53, range 18–88
Gender (male) (%)	49.1	46.0
Provenance of sample (%)		
General practice	33.5	31.0
Inpatient	19.6	26.0
Urology clinic	11.1	8.0
Outpatient clinic (other)	10.8	8.0
Emergency department	7.8	10.0
Antenatal clinic	7.4	12.0
Preoperative	5.1	4.0
Dialysis unit	4.7	1.0

**Figure 1.**
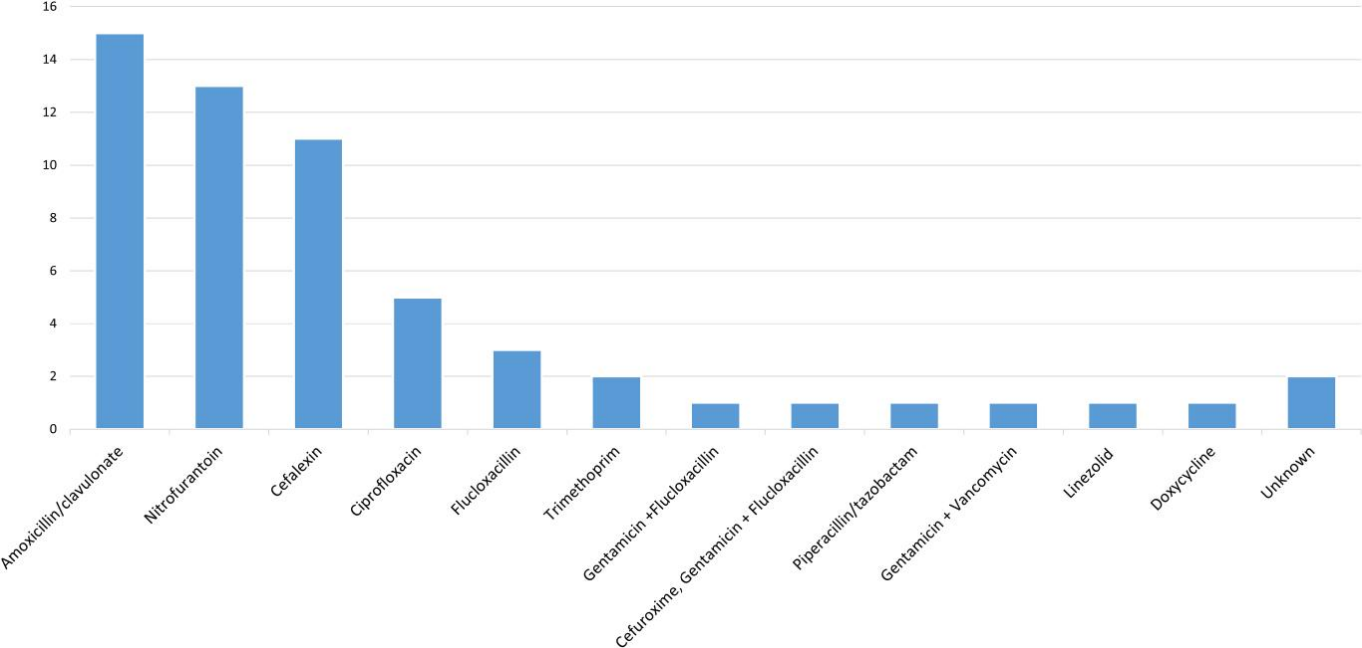
Antibiotics prescribed for the treatment of *S. aureus* bacteriuria (number of cases).

There were 100 urine samples collected in the period between 1 March 2020 and 28 February 2021 and these were studied in greater detail. Demographics for these patients are detailed in Table 1 and indications for urine culture in Table 2; 5% were MRSA. The colony count was typically high (>10^5^ cfu in over half the samples). Microscopy showed large numbers of white cells in 40% of samples, moderate numbers in 17%, and small or insignificant numbers in the remaining 43% of samples. Sixty-three percent of samples were mid-stream urine, 27% were catheter specimens, 3% nephrostomy urine and 7% were unknown. Clinical information from the request or electronic notes was available for 97/100 cases. Of these, 38% of patients were symptomatic with urinary tract infection, and of the 60 patients who had blood tests within 48 h of the urine sample being taken, 28 had raised inflammatory markers. Patients symptomatic with UTI were more likely to have significant pyuria (moderate or large numbers of white cells on microscopy) than those who were asymptomatic (*P* = 0.013). Eighteen percent of patients were known to be diabetic, 39% had urological abnormalities and 17% were pregnant. Fifty-seven percent of patients had recorded antibiotic treatment (Figure 1). Forty-nine percent of patients had a repeat urine culture within 3 months of the positive sample. There was no significant difference in the rate of known recurrence within 3 months of those treated with antibiotics compared with those who were not known to be treated with antibiotics. Recurrence was commoner in inpatients and patients with urological abnormalities or diabetes, although this was not statistically significant. Urinary catheterization was significantly associated with recurrence (*P* < 0.05) (Table 3). Three of the five patients with MRSA SABU had recurrences within 3 months.

**Table 2.** Indications for urine culture as stated on request forms (*n* = 100)

Indication for urine culture indicated on request form	Percentage
Local symptoms of UTI, e.g. frequency, dysuria, lower abdominal pain or loin pain	13
Systemic features consistent with UTI, e.g. fever, delirium, sepsis	12
Screening for asymptomatic bacteriuria	
Pre-urological procedure	19
Pregnancy	9
Other	
Positive urine dip	7
Local infection around catheter/nephrostomy	2
Recurrent UTI	1
Unspecified	37

**Table 3.** Characteristics of cases with (*N* = 13) and without (*N* = 36) recurrence within 3 months, of those who had repeat urine cultures

	Recurrence, *n* (%)	Non-recurrence, *n* (%)	*P* value^a^
Antibiotic treatment	7 (54)	23 (64)	0.524
Male	8 (62)	16 (44)	0.291
Age >65	4 (31)	10 (28)	0.838
Diabetes	4 (31)	4 (11)	0.100
Urological abnormality	8 (62)	14 (39)	0.159
Catheter	7 (54)	7 (19)	0.018*
Inpatient	5 (38)	7 (19)	0.172

a Statistically significant: *P≤0.05.

Of the total 511 cases, only 93 had blood cultures taken within 3 months of the positive urine culture and 6 (6.5%) of these were positive for *S. aureus*. Four of these bacteraemias occurred after urological instrumentation and were thought to be secondary to seeding from bacteriuria. One occurred in a patient with a complex urological history with an enteroureteric fistula, and again the source was felt to be urinary. None of these patients had another source for *S. aureus* bacteraemia identified or had positive cultures from any other site. One patient had SAB secondary to necrotizing pneumonia. All six were inpatients and systemically unwell and all had MSSA bacteraemias. Of note, of the patients who did not have blood cultures taken within 3 months of SABU, none were subsequently diagnosed with SAB within a year at our centre. Sixteen of the 100 patients whose notes were studied in detail had blood cultures taken. Indications were pyrexia (10/16), sepsis (3/16), raised inflammatory markers (2/16) and seizures (1/16). All 16 were inpatients or in the emergency department. None of the 100 patients studied in more detail was found to have any other deep site of infection (2 had positive penile swabs, 1 had positive seminal fluid and 1 had a positive skin swab.)

## Discussion

*S. aureus* is a relatively uncommon isolate in urinary cultures, but prevalence may be increasing.^8^ Risk factors have consistently been found to include urinary tract catheterization, long-term care, hospitalization, older age and comorbidities. Choice of treatment is not always obvious, particularly due to concerns of antibiotic penetration to the target area.

Our service evaluation, conducted at a tertiary centre in the UK, showed a wide range of practice in the investigation and management of SABU. This may in part be linked to the variety of provenance of our samples, which included inpatient and outpatient services, specialist urology clinics and antenatal clinics. Other studies have focused on just one area.^3^ The implications of SABU in these differing populations, with different risk factors for morbidity and mortality associated with SABU, are wide ranging. Just under half of the patients in our evaluation were investigated further following their diagnosis of SABU: 49% had repeat urine cultures taken and 18% had blood cultures taken. This may reflect the high proportion of clinically well, asymptomatic outpatients in the cohort. Thirty-seven percent of patients were symptomatic of urinary tract infection but 57% were treated with antibiotics, implying that there was treatment of asymptomatic bacteriuria, despite the low levels of investigation. It is possible this reflects a lack of awareness among treating clinicians of the differing implications of colonization, UTI and bacteriuria potentially associated with bacteraemia, in patients with different risk factors.

In this evaluation only 6 of 511 patients with SABU had proven SAB within 3 months, although only 93 had blood cultures taken, so it is possible that there were unidentified bacteraemias. This lower level of known bacteraemia compared with previous studies may be due to several factors. First, the majority of patients were outpatients under the age of 65, making them lower risk for SAB than the cohort studied by Muder *et al.*,^11^ for example, who were residents in long-term care. Second, as noted, only 18% of patients had blood cultures taken. The majority of patients were outpatients, including patients seen by community general practice, where it is not possible to order blood cultures. Third, MRSA made up only 6.5% of the SABU cases detected. This is in contrast to other studies, particularly from the USA, where MRSA isolates make up almost half of SABU cases.^14^ MRSA SABU appears to be more strongly associated with SAB than MSSA SABU.^14^

Four of the six cases of bacteraemia in our cohort occurred after urological instrumentation in patients with SABU. Bacteraemic seeding in such contexts is well recognized^16^ and suggests that pre-emptive antibiotic treatment in patients prior to instrumentation is warranted.

Recurrence of SABU was linked to urinary catheterization, urinary tract abnormality, diabetes and inpatient status. It is recognized that persistent colonization of urine with *S. aureus* is a risk factor for infection,^11^ and that in such patients measures such as decolonization, antibiotic treatment and avoiding catheterization, may be beneficial.

Antenatal clinics accounted for 7.4% of urine samples positive for *S. aureus*. It is recommended that pregnant women with asymptomatic bacteriuria should be treated, in order to reduce preterm birth and postnatal pyelonephritis.^17,18^ In our evaluation 91% had been treated with an appropriate antibiotic course.

**Figure 2.**
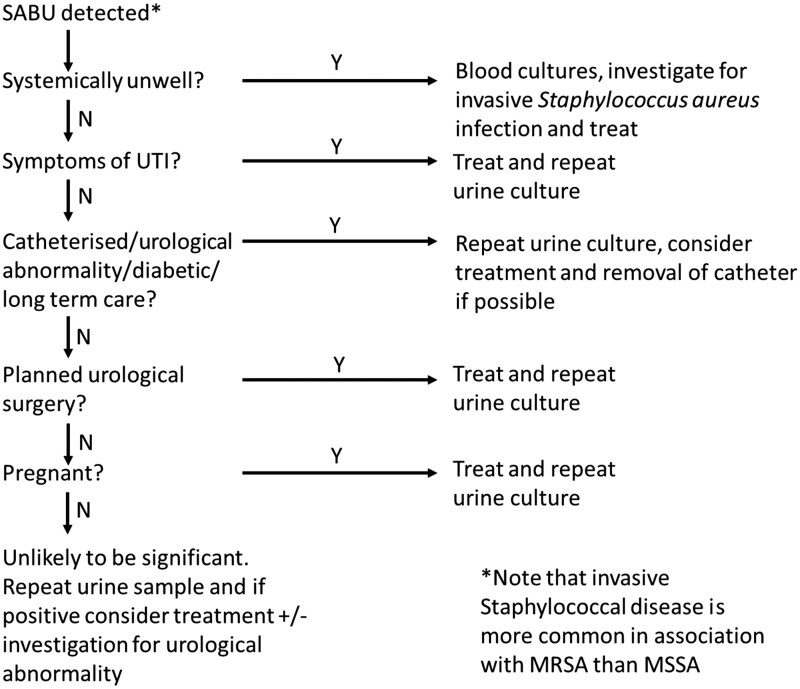
Proposed algorithm for the investigation and management of SABU. ‘Systemically unwell’ may include patients with fever, raised inflammatory markers or needing acute admission. The term is broad to allow for physician judgement, and there should be a low threshold for considering these investigations. N, no; Y, yes.

There is a lack of evidence to support treating asymptomatic SABU with antibiotics. Although it has previously been proposed that all cases of SABU should prompt investigations with blood cultures,^5,8^ we suggest based on our data and in agreement with Karakonstantis and Kalemaki^10^ that in low-risk patients with asymptomatic SABU and no indication of invasive staphylococcal infection further investigation or treatment may not be necessary. We therefore propose an algorithm for the investigation and management of SABU in our centre (Figure 2).

Our proposed algorithm has a few differences from that proposed by Karakonstantis and Kalemaki.^10^ First, we do not suggest waiting for a second sample before initiating investigations in patients who may be unwell, as this could lead to delay in diagnosis of severe disease such as infective endocarditis.^6^ Second, we have added a category for those who may be at higher risk of persistent colonization and later SAB. Third, we have specifically highlighted two categories of patients (pregnant women and patients planned to have urological instrumentation), in whom treatment of SABU, even if asymptomatic, may prevent future severe infection.

Antibiotic choice should be based on local sensitivity patterns. In our evaluation there was a low level of resistance to usual first-line antibiotics.

Microbiology comments on reports of urine cultures positive for *S. aureus* can be useful in guiding clinicians.^19^ An appended comment suggesting repeat culture or blood cultures if the patient is unwell, or referring the clinician to a local guideline, and with prompts such as highlighting the need to treat in pregnancy, could be the basis for future work and service improvement.

Our analysis has several limitations. The sample size, although comparable to other studies of SABU, is small. Notes were not always complete or available, resulting in incomplete clinical data. As this was a restrospective evaluation, urine samples were taken from a variety of clinical situations, which may not reflect the wider population. Finally the low rates of repeat urine cultures and blood cultures mean we cannot draw robust conclusions of rates of recurrence and bacteraemia. Locally, we will introduce the above algorithm and alter our reporting to reflect this, and will re-evaluate our management following these changes. Larger prospective studies of both inpatient and outpatient populations are needed to further inform investigation and treatment of SABU.

### Conclusions

Our evaluation suggests that investigation and management of *S. aureus* bacteriuria is variable. Although this is a small cohort, it is comparable to other cohorts studied. The majority of samples taken were from outpatients and most were asymptomatic and had normal inflammatory markers. Our experience demonstrates the range of implications of SABU including as an indicator of deep-seated staphylococcal infection or SAB. However, it does not support the routine taking of blood cultures or treatment of asymptomatic bacteriuria in well patients with *S. aureus* bacteriuria in this setting. Repeat urine culture, and investigation and treatment of higher risk patients, for example prior to bladder instrumentation, may be warranted. Comments appended to the microbiology result may be useful in guiding clinicians and we would propose incorporation of this in reporting pathways.
